# Increased Lipids in *Chlamydomonas reinhardtii* by Multiple Regulations of DOF, LACS2, and CIS1

**DOI:** 10.3390/ijms231710176

**Published:** 2022-09-05

**Authors:** Bin Jia, Jianbo Yin, Xiaolian Li, Yingling Li, Xingcai Yang, Chengxiang Lan, Ying Huang

**Affiliations:** Guangdong Technology Research Center for Marine Algal Bioengineering, Guangdong Provincial Key Laboratory for Plant Epigenetics, Shenzhen Engineering Laboratory for Marine Algal Biotechnology, Longhua Innovation Institute for Biotechnology, College of Life Sciences and Oceanography, Shenzhen University, Shenzhen 518060, China

**Keywords:** DOF-type transcription factor, long-chain acyl-CoA synthetase, citrate synthase, gene overexpression, antisense knockdown, transcriptome analysis, triacylglycerol, *Chlamydomonas reinhardtii*

## Abstract

Microalgal lipids are essential for biofuel and dietary supplement production. Lipid engineering for higher production has been studied for years. However, due to the complexity of lipid metabolism, single-gene engineering gradually encounters bottlenecks. Multiple gene regulation is more beneficial to boosting lipid accumulation and further clarifying the complex regulatory mechanism of lipid biosynthesis in the homeostasis of lipids, carbohydrates, and protein metabolism. Here, three lipid-related genes, DOF, LACS2, and CIS, were co-regulated in *Chlamydomonas reinhartii* by two circles of transformation to overexpress DOF and knock down LACS2 and CIS simultaneously. With the multiple regulations of these genes, the intracellular lipids and FA content increased by 142% and 52%, respectively, compared with CC849, whereas the starch and protein contents decreased by 45% and 24%. Transcriptomic analysis showed that genes in TAG and FA biosynthesis were up-regulated, and genes in starch and protein metabolism were down-regulated. This revealed that more carbon precursor fluxes from starch and protein metabolism were redirected towards lipid synthesis pathways. These results showed that regulating genes in various metabolisms contributed to carbon flux redirection and significantly improved intracellular lipids, demonstrating the potential of multiple gene regulation strategies and providing possible candidates for lipid overproduction in microalgae.

## 1. Introduction

Microalgae lipids show great potential in biorefinery for edible oil, functional foods, biofuel, and animal feed production [1,2]. Lipid metabolism is a very sophisticated and dynamic metabolic process that involves several pathways, including fatty acid (FA) synthesis, TAG synthesis, and FA oxidation, which are interconnected. For example, FAs can directly participate in TAG synthesis as side chains, and they can also be oxidized to provide acyl-CoAs as substrates for lipid biosynthesis. In addition, lipid metabolism is closely associated with carbohydrate and protein metabolism to establish a complex regulation network of carbon and nitrogen substance metabolism [3].

*C. reinhardtii*, a model photoautotrophic microalga, is a promising material for transgenic engineering because of its high photosynthetic efficiency, fast growth rate and diverse transformation systems [4]. Lipid engineering for higher production has been widely studied in *C. reinhardtii* regarding FA synthesis, Kennedy pathway, transcription factors, and NADPH generation [5]. FA synthesis, the first step of lipid formation, occurs in the chloroplast of *C. reinhardtii* using acetyl-CoA as a substrate. Acetyl-CoA carboxylase (ACC), malonyl-CoA ACP transacylase (MAT), 3-ketoacyl-ACP synthase (KAS), 3-ketoacyl-ACP reductase (KAR), 3-hydroxyacyl-ACP dehydrase (HD), and enoyl-ACP reductase (ENR) are the key enzymes involved in this process [5]. Glycerolipids are synthesized stepwise in the Kennedy pathway through the catalysis of glycerol 3-phosphate acyltransferase (GPAT), lysophophatidic acid acyltransferase (LPAT), phosphatidic acid phosphatase (PAP), and diacylglycerol acyltransferase (DGAT). Because transcription factors can control the expression of multiple genes, recently, they were considered as important targets for lipid engineering. Transcription factor crDOF [6], CrMYB1 [7], and BZIP1 [8] in *C. reinhardtii* were demonstrated to be associated with lipid synthesis.

Efforts have been made in the overexpression of key genes in FA synthesis, Kennedy pathway or transcription factors, and by the knockdown of competing pathway genes to enhance lipid production. For example, overexpressing an endogenous DOF transcription factor (CrDOF) regulates carbon and nitrogen substance metabolism in plant cells, resulting in a total of 126.01 μg/mg of intracellular FAs that increased by 23.24% in *C. reinhardtii* [6]. Similarly, overexpression of soybean DOF increased the total lipids by 46.4~52.9% and 1.8~2.3-fold in *Chlorella ellipsoidea* and *C. reinhardtii*, respectively [9,10]. Overexpression of CrDGTT4 in *C. reinhardtii* resulted in a 2.5-fold increase in TAG during phosphate starvation condition [11]. On the other hand, knockdown of long-chain acyl-CoA synthetase (LACS2) which can active FA into CoA thioesters increased the lipid content by 55% [12]. Knockdown of the citrate synthase (CIS1), a rate-limiting enzyme in the tricarboxylic acid cycle which is predicted to be located in the mitochondria [13], rose the TAG by 169.5% in *C. reinhardtii* [14]. 

Due to the complexity of the lipid metabolism network, multiple gene regulation always performs better than regulation of a single gene. For example, co-expression of *GPAT1* and *LPAT1* increased TAG content 2.3 fold, and co-expression of *GPAT* and *DGAT2* caused a 3.6-fold increase in total lipids in *Phaeodactylum tricornutum* [15]. Additionally, combined overexpression of *GPAT, LPAT*, and *DGAT* in *Neochloris oleoabundans* led to a 4.5-fold increase in TAG [16]. However, multiple gene regulation of lipid biosynthesis in *C. reinhardtii* is still scarce for technical reasons. 

In this study, multiple gene regulations in lipid, carbohydrate, and protein metabolism were conducted by iterative circles of gene transformations to redirect the intracellular carbon fluxes. Furthermore, transcriptome analysis was used to elucidate the effect of multiple regulations on lipid metabolism networks and to provide potential gene candidates for industrial application of microalgal lipid production.

## 2. Results

### 2.1. Co-Regulation of DOF, LACS2, and CIS1 in C. reinhardtii 

Co-regulation of several genes in *C. reinhardtii* is challenging work, but it can be a promising method for lipid accumulation due to its complicated metabolic network. Referring to earlier publications, we focused on overexpression of *crdof* and knockdown of *cracs2* and *cis1* for rational control of lipid metabolism [6,12,14]. To achieve this, iterative circles of gene transformations were conducted. Endogenous DOF was transformed into wild type (WT) in the first circle to generate the DOF strain. In the second circle, the *cracs2* and *cis1* of the DOF strain were silenced by RNAi plasmid pMaa7 XIR/LACS2-CIS1IR. Finally, 150 strains were randomly picked, cultured, and stained by BODIPY. Algae strains showed varied fluorescence intensities because of the random integration of exogenous genes in *C. reinhardtii*. Among them, 33 strains exhibited higher fluorescence intensities than WT (Figure S1). After PCR verification, eight strains showed expected results. The top three strains (DLC-4, 7, and 8) with high fluorescence intensities were subjected to qRT-PCR analysis (Figure S2). Only the mRNA expression levels of CIS and LACS2 in DLC-4 showed decreased patterns. Thus, DLC-4 was selected for further analysis. 

**Figure 1:**
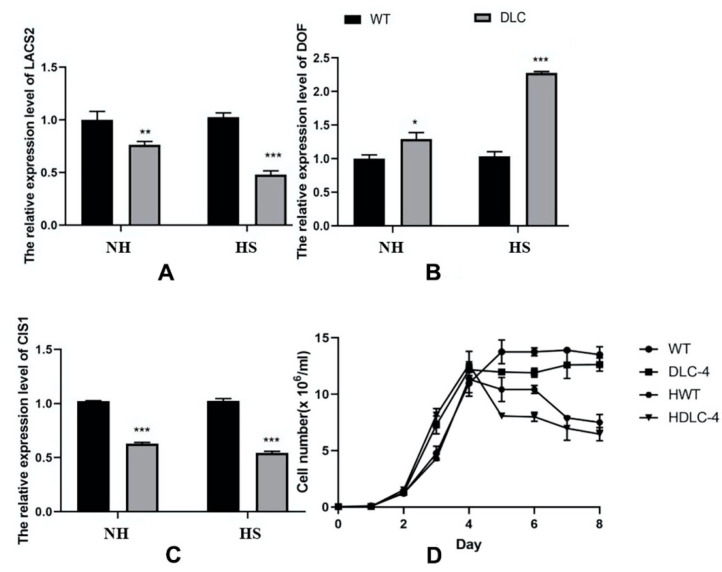
The mRNA expression patterns of *crdof, cracs2,* and *cis1* in the DLC-4 and WT when strains were treated with HS (HS) and without HS (NH). (**A**) The expression levels of LACS2. (**B**) The expression levels of DOF. (**C**) The expression levels of CIS1. Statistical significance (one-way ANOVA) is represented by asterisks (*, **, and *** indicate a difference at *p* ≤ 0.05, *p* ≤ 0.01. and *p* ≤ 0.005, respectively). (**D**) Growth curves of the DLC-4 and WT treated with heat shock (HDLC-4 and HWT) or without heat shock (DLC-4 and WT) at the mid-log phase. Error bars depict SD.

DLC-4 showed slightly lower growth and cell density at the stationary phase than WT (Figure 1D). The transcription abundances of *cracs2, cis1,* and *crdof* in DLC-4 were determined and are shown in Figure 1. The mRNA levels of *crdof* increased by 29%, whereas those of *cracs2* and *cis1* were reduced by 23.8% and 38.5%, respectively, compared with those of WT. After heat shock (HS), the mRNA levels of *crdof* rose by 121%, whereas those of *cracs2* and *cis1* decreased by 53.3% and 46.9%. These results showed that two transformation circles had achieved multiple gene regulation in *C. reinhardtii*. As expected, the overexpression of *crdof* and knockdown of *cracs2* and *cis1* were completed in transgenic strain DLC-4.

### 2.2. Lipids, Carbohydrate, and Protein Variety in DLC Strains

**Figure 2:**
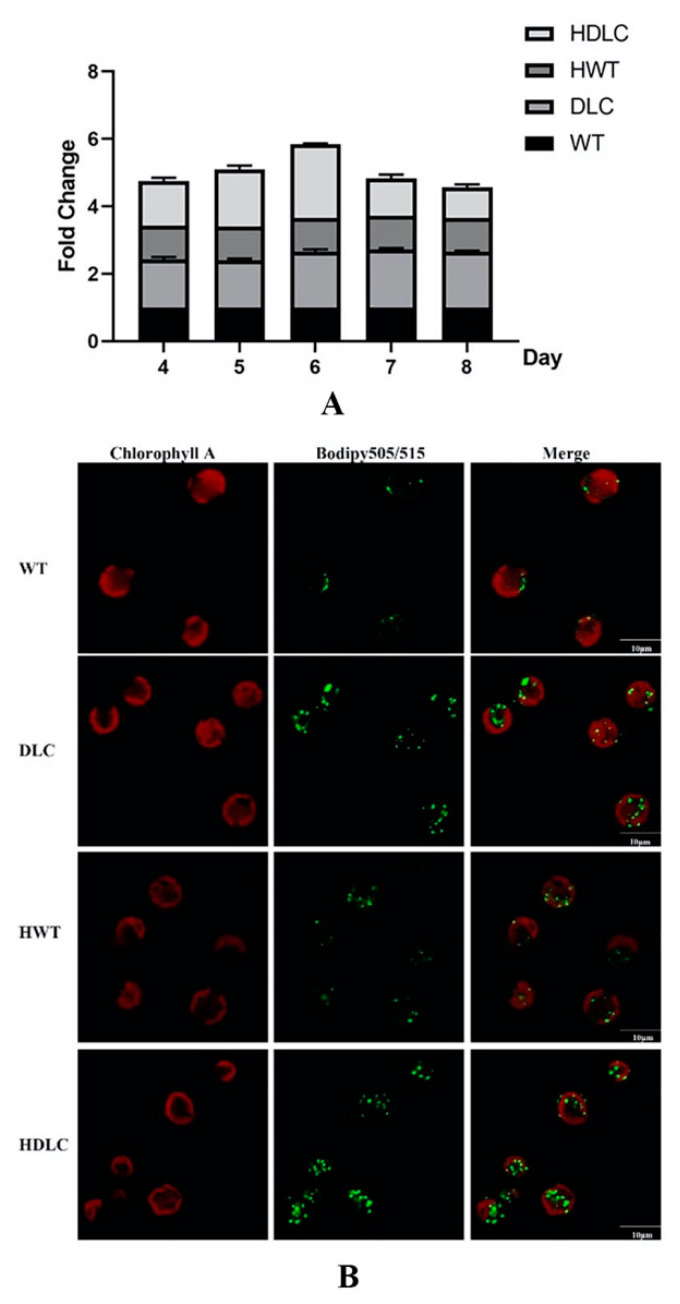
Intracellular neutral lipid contents in the DLC-4 and WT when they were treated with HS (HDLC and HWT) or without HS (DLC and WT) at the mid-log phase. (**A**) The lipid profile of strains from day 4 to day 8. Results are shown as the fold changes of the fluorescence intensities of strain DLC-4 compared with those of the WT strain after BODIFY staining. Error bars depict SD. (**B**) The distribution of intracellular neutral lipids in the DLC-4 and WT. Lipid droplets were stained by BODIFY505/515 and visualized by a confocal microscope. Each scale bar indicates 10 μm.

With the success of multiple gene regulations in DLC strains, it is necessary to further analyze their intracellular metabolite changes. Consequently, intracellular neutral lipid profiles of the DLC-4 strain from day 4 to day 8 with or without HS treatment were analyzed. As shown in Figure 2A, the DLC-4 strain showed obvious higher levels of neutral lipids than WT throughout the whole culture time, regardless of whether it was HS treated. Additionally, the neutral lipids of all strains increased gradually and reached a peak on day 6. Thus, a detailed lipid analysis was conducted on day 6. At that time, the neutral lipids of the DLC-4 strain showed a 1.2-fold and 0.7-fold increase over those of WT, respectively, when strains were treated by HS and not. Meanwhile, the TAG droplet distributions of strains were observed by confocal microscope and are shown in Figure 2B. The number and size of lipid droplets in the DLC-4 were both remarkably increased compared with those in WT. Significantly, the DLC-4 presented the strongest fluorescent signal after HS treatment.

**Figure 3:**
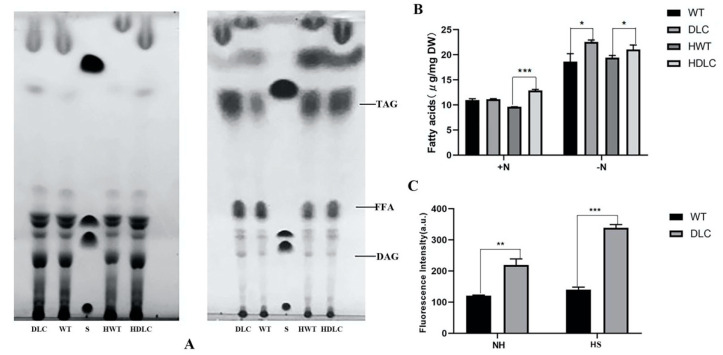
Analysis of the TAG in the DLC-4 and WT when they were treated with HS (HDLC and HWT) or without HS (DLC and WT). (**A**): Lipids were separated on a TLC plate and visualized with iodine. The lipids from strains in the nitrogen-replete and nitrogen-deprived conditions are shown separately in the left and right panels. (**B**): The TAG content of the DLC-4 and WT separated from TLC. +N: nitrogen-replete condition, −N: nitrogen-deprived condition. (**C**): The TAG content of DLC-4 and WT under nitrogen-deprived conditions when strains were treated with HS (HS) and without HS (NH). TAG was stained by BODIPY and measured by a microplate reader. Statistical significance (one-way ANOVA) is represented by asterisks (*, **, and *** indicate a difference at *p* ≤ 0.05, *p* ≤ 0.01, and *p* ≤ 0.005, respectively). Error bars depict SD.

Microalgae prefer to accumulate more lipids under nutrient-deprivation conditions (ND). Thus, the intracellular lipids of strains under ND were further separated through TLC and quantified by GC-MASS. As expected, the TAG and FA bands of strains under ND were substantially larger than those of strains under normal conditions in the TLC plate (Figure 3A) Additionally, the TAG and FA bands of the DLC-4 were larger than those of WT under both normal conditions and ND. This indicated that the DLC-4 synthesized more TAG and free FAs than WT. The TAG fraction analysis by GC-MASS also showed that HDLC-4 displayed 8.3% and 21% more TAG than the WT with HS and without HS, respectively (Figure 3B). In addition, TAG contents of strains were also determined by BODIPY staining. The DLC-4 exhibited 82% higher fluorescence intensities than WT under ND. After HS, the DLC-4 showed 142% higher fluorescence intensities than WT (Figure 3C). These results suggested that multiple gene regulation of DOF, LACS2 and CIS1 in *C. reinhardtii* significantly improved TAG content.

**Figure 4:**
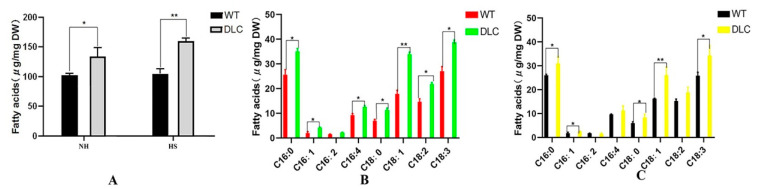
Total FA and lipid profiles in the DLC-4 and WT. (**A**) Total FAs of the DLC-4 and WT when strains were treated with HS (HS) and without HS (NH). (**B**) The profiles of the total lipids in the DLC-4 and WT when strains were treated with HS. (**C**) The profiles of the total lipids in the DLC-4 and WT when strains were treated without HS. Statistical significance (one-way ANOVA) is represented by asterisks (* and ** indicate a difference at *p* ≤ 0.05 and *p* ≤ 0.01, respectively). Error bars depict SD.

Total intracellular FA profiles were also analyzed by GC-MASS. The total FA of the DLC-4 and WT were 159.9 mg/g and 105.0 mg/g, and 133.9 mg/g and 102.6 mg/g dry cell weight (DCW) when strains were treated with and without HS, respectively. Thus, the titer and productivity of DLC-4 for lipids were 187.1 mg/L, and 32.2 mg/(L·d). Compared with WT, the total lipids of the DLC-4 increased by 52% and 31% with and without HS treatment, respectively (Figure 4A). However, the major lipids were still C16:0, C18:1, and C18:3 (Figure 4B,C). Moreover, the unsaturated FAs of the DLC-4 rose by 34% and 57% compared with WT when they were treated with HS and without HS, respectively. The content of FAs C16:1, C18:1, and C18:3 was increased significantly (*p* < 0.01) in the DLC-4, with increases of 48%, 60%, and 33%, respectively, without HS. Similarly, the content of FAs C16:1, C16:4, C18:1, C18:2, and C18:3 increased significantly (*p* < 0.01) in the DLC-4, with increases of 104%, 36%, 90%, 49%, and 43%, respectively after HS. These data indicate that multiple gene regulation promoted the accumulation of the unsaturated FAs and the total lipids in *C. reinhardtii*.

Carbohydrate, protein, and lipid metabolisms are closely connected to provide energy for the organism. As exhibited in Figure S3, after HS, the starch and protein contents in DLC-4 were 61.9 mg/g and 124.6 mg/g DCW, respectively, which showed a 45% and 24% decrease compared with those in WT, respectively. At the same time, there was no significant difference in total carbohydrate content. These changes indicated the redirection of carbon flux in carbohydrate and protein metabolism.

### 2.3. Differentially Expressed Genes (DEGs) in DLC Strain

To discover the molecular mechanism of the multiple gene regulation in algal cells, transcriptome analysis of DLC-4 and WT after HS treatment was conducted. Two libraries were generated, and RNA sequencing was performed on the BGISEQ-500 platform. In this step, 49.19 and 49.77 million raw reads were obtained for WT and DLC-4, respectively. After filtering the raw data, 43.48 and 44.33 million clean reads were mapped to the *C. reinhardtii* reference genome. Approximately 93.02% of the sequences of WT could be mapped to the genome, and nearly 90% of the sequences had unique alignment positions on the reference genome; it was the same case with DLC-4. In both strains, 17379 genes were identified, among which 2756 genes were differentially expressed. Compared with WT, the number of up- and down-regulated DEGs in DLC-4 were 2130 and 626, respectively.

### 2.4. Distributions of DEGs among KEGG Pathways

**Figure 5:**
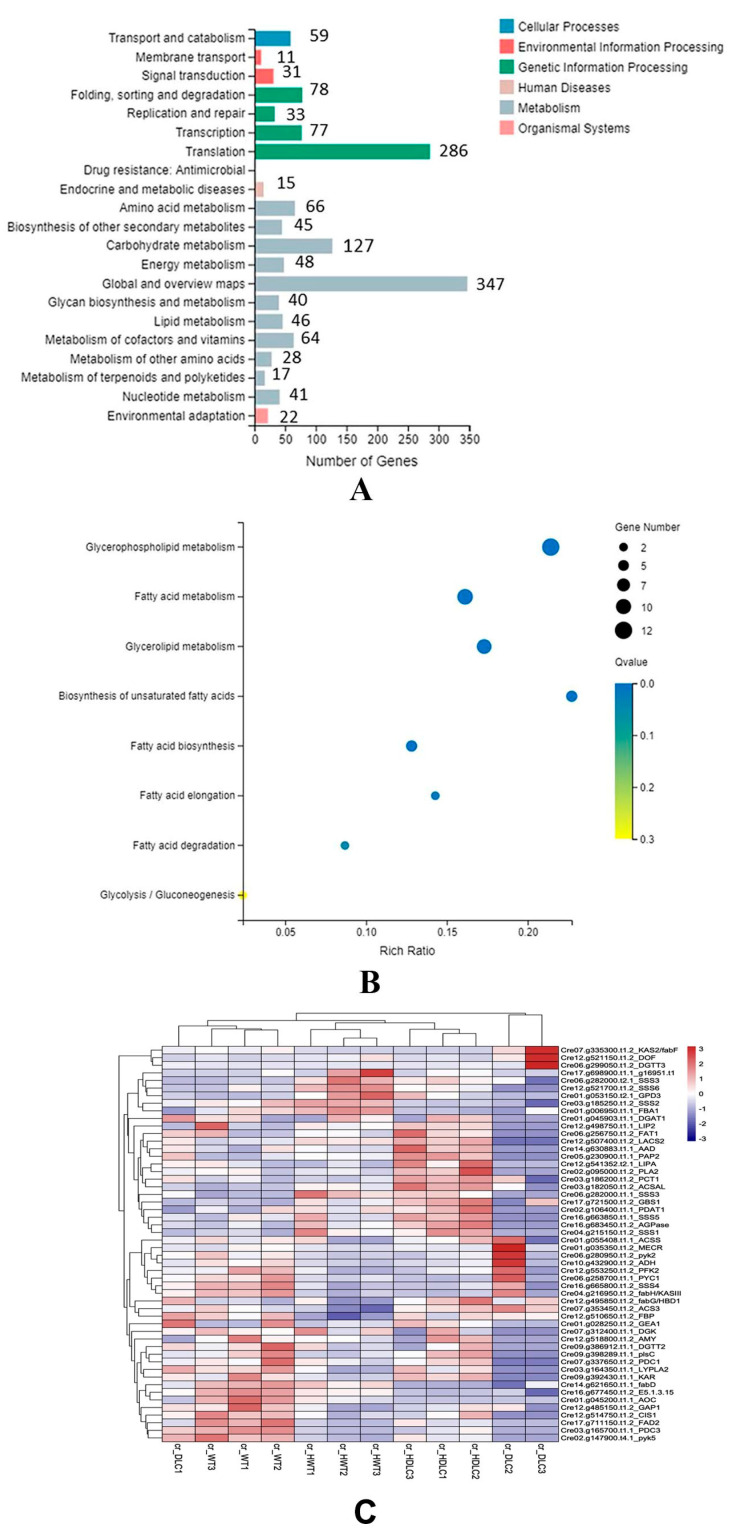
KEGG pathway classification and enrichment of DEGs from DLC vs. WT. (**A**) KEGG pathway classification of DEGs. (**B**) KEGG enrichment of DEGs involved in the lipid metabolism. The Q value ranges from 0 to 0.3. A Q value closer to zero suggests more considerable enrichment. Pathways with Q values ≤ 0.05 were defined as significantly enriched pathways for the DEGs. (**C**) The clustering heatmap of DEGs related to lipid metabolism. For the clustering heatmap, normalized counts were rescaled between −3 and 3; clustering was based on Pearson correlation.

KEGG pathway classification indicated that the DEGs were clustered into 21 KEGG pathways. The top five metabolism pathways containing the most DEGs were carbohydrate metabolism (127 DEGs), amino acid metabolism (66 DEGs), metabolism of cofactor and vitamins (64 DEGs), energy metabolism (48 DEGs), and lipid metabolism (46 DEGs, Figure 5A). KEGG enrichment analysis of lipid metabolism showed that DEGs were enriched in the biosynthesis of unsaturated FAs, glycerophospholipid metabolism, glycerolipid metabolism, FA metabolism, FA elongation, and FA degradation (Figure 5B). To further understand the metabolic flux changes resulting from multiple gene regulations in the DLC-4, DEGs enriched in FA metabolism, triacylglycerol biosynthesis, and carbohydrate metabolism were focused on.

DEGs involved in the synthesis of acetyl-CoA, the main building block for de novo FA synthesis, are pyruvate decarboxylase (PDC), phosphofructokinase (PFK), pyruvate kinase (PYK), and pyruvate carboxylase (PYC). The PDC, PFK, and PYK are up-regulated, and PYC is down-regulated in the DLC-4. In the FA synthesis pathway, critical genes in the FA synthase complex, such as β-ketoacyl-acyl carrier protein synthase (KASI and KASII) and 3-ketoacyl-acyl carrier protein reductase (KAR), were up-regulated in the DLC-4. In the TAG synthesis pathway, phospholipase A2 (PLA2), lysophospholipase A2 (LYPLA2), phosphatidic acid phosphatase 2 (PAP2), diacylglycerol acyltransferase (DGAT), and phospholipid: diacylglycerol acyltransferase (PDAT) were all also up-regulated in the DLC-4. Meanwhile, Δ12 fatty acid desaturase and acyl carrier protein desaturase (ADD) involved in unsaturated FA synthesis were up-regulated in DLC-4. Furthermore, several DEGs converting TAG into free FAs, such as triacylglycerol lipase 2 (LIP2) and glycerol-ester acyl hydrolase 1 (GEA1), were found up-regulated in the DLC-4. This potentially enhanced the recycle rate of FAs, therefore profoundly accelerating the redirection of carbon flux towards lipid metabolism.

Four starch synthase (SS) genes were down-regulated in the carbohydrate metabolism, and α-amylase (α-AMY), crucial for amylolysis, was up-regulated in HDLC-4. Furthermore, *cis1* was down-regulated in support of the knockdown of *cis1* in HDLC-4. These data indicate that carbohydrates, predominantly starch, were inhabited under the control of multiple gene regulation. In amino acid metabolism, branched-chain amino acids (BCAA) play an essential role in the homeostasis of TAG biosynthesis [17]. Five BCTA genes involved in BCAA catabolism were up-regulated in HDLC-4.

As mentioned above, the results of transcriptomic analyses provided applicable proof to indicate that more carbon precursors were redirected towards lipid metabolism from carbohydrate and protein metabolism under the regulation of DOF, LACS2, and CIS1, in accordance with the notable decrease in starch and protein content and remarkable improvement in TAG and FA content (Figure 3).

### 2.5. The Expression Levels of DEGs: Real-Time Quantitative PCR

**Figure 6:**
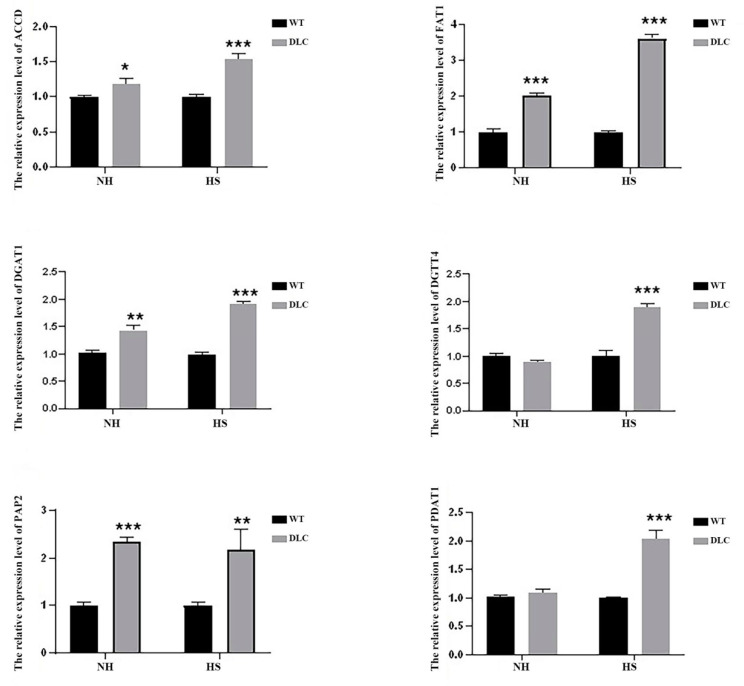
The qRT-PCR analysis of the expression levels of key genes involved in lipid metabolism in the DLC-4 and WT when strains were treated with HS (HS) and without HS (NH). Statistical significance (one-way ANOVA) is represented by asterisks (*, **, and *** indicate a difference at *p* ≤ 0.05, *p* ≤ 0.01, and *p* ≤ 0.005, respectively). Error bars depict SD.

To validate the DEGs data, the expression levels of six genes (ACCD, FAT1, DGAT1, DGTT4, PAP2, and PDAT1) of DLC-4 and WT were analyzed by qRT-PCR. Under HS treatment, the expression levels of all genes in DLC-4 showed a significant increase compared with those in WT (Figure 6). Our transcriptome analysis is consistent with this qRT-PCR, indicative of the reliability of the transcriptome data. Furthermore, when strains were not treated with HS, the expression levels of ACCD, FAT1, DGAT1, and PAP2 in the DLC-4 were also higher than those in WT. These data indicate that the effects of multiple gene regulation are relatively stable.

## 3. Discussion

Gene overexpression, gene knockdown, and gene knockout are three conventional gene regulation modes which have already been performed in *C. reinhardtii.* However, studies are still focused on single-mode regulation or single-gene regulation. Multiple-gene regulations and multiple-mode regulations are still sometimes challenging. In terms of gene regulation mode, gene overexpression has been well developed with diversified tool boxes [18], and gene knockdown based on artificial miRNA [19] or RNAi has also been widely used [20]. However, gene knockout needs to be improved. The knockout efficiency is not high, and the available target gene is limited [21,22]. When further considering the number of regulations of genes, multiple gene overexpression is common. Using 2A peptide [23] or tandem expression cassettes, 2–4 genes can be normally expressed [24]. Multiple-gene knockouts need to be developed. Co-knockout of wdtc1 and ppx1 has only recently been reported [25]. Multiple-gene knockdown using tandem inverted repeats can achieve co-knockdown of two genes [26]. This paper used two rounds of iterative transformation to co-regulate three genes in two modes. In the first round, DOF was overexpressed; in the second round, CIS and LACS2 were knocked down by tandem inverted repeats. This strategy can couple gene regulation modes and increase the number of regulations of genes, and provide useful technology for complex metabolic pathway study.

DOF, LACS2, and CIS are involved in multiple metabolic pathways in *C. reinhardtii*. Knockdown of LACS2, a gene involved in β-oxidation, increased the total FA by 45% to 130 mg/g DCW [12]. Silencing the CIS gene could up-regulate PAP2 and DGAT and increase TAG accumulation by 169.5% [14]. Overexpression of DOF could increase total FAs by 23% to 125 mg/g DCW. The neutral lipid measured by BODIPY staining increased twofold [6]. In this study, CIS and LACS2 were silenced, and DOF was overexpressed simultaneously. Multiple gene regulation reduced protein and starch contents by 24.0% and 45%, respectively. In comparison, the contents of total FAs increased by 52% to 159.9 mg/g, and the neutral lipid increased by 142%. The content of intracellular unsaturated fatty acids also increased significantly; for example, the content of c16:1 increased by 104%. These data suggested that multiple regulations have effects on single regulations.

Starch and lipids, two energy storage substances of *C. reinhardtii*, compete on carbon flow with the same carbon precursor glyceraldehyde-3-phosphate (G3P). The metabolisms of starch and lipid biosynthesis are highly related to each other. In ND condition, *C. reinhardtii* preferred to accumulate starch in the early stage of treatment and lipids in the middle and late stage of treatment [27,28]. Thus, starch is thought to be degraded to contribute to the carbon source for lipid accumulation, and enzymes related to starch degradation, such as amylase and starch phosphorylase, can trigger the lipid synthesis [29,30]. Here, transcriptome analysis showed that the mRNA levels of genes in starch metabolism in the DLC-4 were changed. For example, the expression levels of the starch synthesis gene (SS) decreased, and the expression levels of the α-amylase increased. In addition, the starch in DLC-4 showed a 45% decrease. These data indicate the carbon flow from starch to lipid. Recently, some transcription factors were found to control the switches between starch and lipid synthesis [31]. Phosphorus Starvation Response 1 (PSR1) transcription factor was demonstrated to be a lipid to starch switch. Overexpression of PSR1 can increase the starch but reduce the lipid [32]. Meanwhile, the DOF transcription factors were reported be a starch to lipid switch, which can enhance the lipid but reduce starch [6,33]. Here, after overexpression of crDOF and knockdown of CIS1 and LACS2 in DLC-4, the changes of lipid and starch in this strain showed similar results to the previous study. Meanwhile, the transcriptomic data and the qRT-PCR both showed that ACCD, FAT1, DGAT1, and PAP2 in the DLC-4 were unregulated. These genes were also found to be unregulated in the strains overexpressing DOF alone [6,33]. Thus, we hypothesize that ACCD, FAT1, DGAT1, and PAP2 maybe the target genes of DOF. In addition, branched-chain amino acids (valine, leucine, isoleucine, etc.) can provide precursors and ATP for TAG synthesis [17], and transcriptome analysis revealed a rise in genes for branched-chain amino acid synthesis, indicating that there may be more branched-chain amino acids in the DLC-4 to support TAG synthesis. The above results show that the combinational regulation changed the carbon flow of engineered algae, and more energy and carbon flow were devoted to the synthesis of the lipids.

There are two TAG synthesis pathways in *C. reinhardtii*; one is catalyzed by DGAT using acyl-CoA and diacylglycerol, and the other is catalyzed by PDAT using phospholipids and diacylglycerol. Two types of DGAT, DGATI and DGAT II, containing one gene (DGAT1) and five genes (DGTT1-DGTT5), respectively, were found in *C. reinhardtii* [34]. DGTT1, DGTT2, and DGTT 3 showed little effect on lipid synthesis, whereas DGAT1 and DGTT4 could increase TAG [35]. The transcriptome and qRT-PCR results after HS also showed that the expression of these two genes increased significantly. We also found that the content of C18:1 increased by 36%, possibly caused by the increase in DGTT4 expression due to C 18:1 being the preferred substrate of DGTT4 [11].

**Figure 7:**
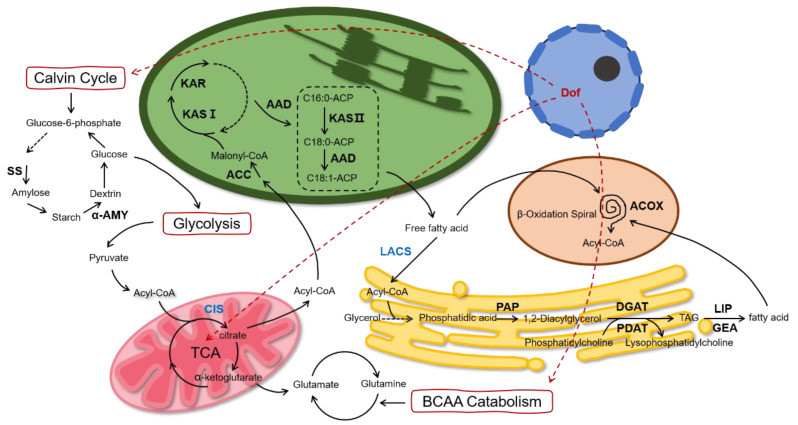
Schematic representation of the multiple regulations of DOF, LACS2, and CIS1.

Based on the above data, we proposed the schematic diagram of lipid metabolism of *C. reinhardtii* after multi-gene regulation (Figure 7). We believe that the intracellular carbon flow is altered under the co-regulation of the above genes. On the one hand, more carbon flows towards FA de novo synthesis and the TAG assembly. On the other hand, the carbon flow to starch and protein synthesis is reduced, causing a decrease in the intracellular starch and protein.

## 4. Materials and Methods 

### 4.1. Strain, Vectors, and Culture Condition

*C. reinhardtii* CC849 was obtained from the Chlamydomonas Resource Center and used as WT strain. *E. coli* Trans-T1 was used for regular DNA manipulation. Vector pJD-crdof was used for DOF gene overexpression with HSP70A-RBCS2 promoter. Vector pMaa7 XIR/LACS2-CIS1IR was applied to conduct RNAi of LACS2 and CIS1 genes [36]. Two vectors were transformed into WT to perform multiple regulations, and the successful transformant was named DLC.

Algae were cultured in tris-acetate-phosphate (TAP) medium under continuous light (60 μmol m^−2^ s^−1^) at 25 °C. Nitrogen deprivation was performed in a TAP-N medium (KCL substituted for NH_4_CL) when cells were grown to the mid-logarithmic phase. To induce overexpression of *crdof*, the DLC-4 was grown to 1 × 10^5^ cells/mL in TAP and subjected to heat shock (40 °C for 30 min). Then cells were continuously cultured at 25 °C for another 48 h.

### 4.2. Plasmid Construction and Transformation

The DOF, LACS2, and CIS genes used in this paper were cloned using primers listed in Table S1. DOF expression plasmid pJD-crdof was constructed by inserting *crdof* into vector pJD-luc under the control of the HSP70A-RBCS2 promoter. LACS2- and CIS1-silencing plasmid pMaa7 XIR/LACS2-CIS1IR was created by concurrent reverse insertion of *cracs2*, *cis1*, and the 3′-untranslated region of *Maa7* into vector pMaa7 XIR under the control of the RBCS2 promoter. Transformation of plasmids pJD-crdof and pMaa7 XIR/LACS2-CIS1IR into *C. reinhartii* was performed by the glass bead agitation method. Hygromycin (10 μg/mL), L-Tryptophan (1.5 mmol/L), and 5-fluoroindole (5 μmol/L) were applied to the selection of the transformants. To validate successful transformants, genomic DNA from the algae cells was extracted and used as a template for PCR amplification of the LACS2-CIS1 fragment in the pMaa7 XIR/LACS2-CIS1IR vector. 

### 4.3. Quantitative Reverse Transcription-PCR (qRT-PCR)

Total RNA was isolated by Takara RNAiso Plus Kit according to its instructions, and cDNA was synthesized by Takara Reverse Transcriptase M-MLV using oligo-dT as the reverse primer according to its protocol. With KOD SYBR Qrt-PCR Mix (TOYOBO), one μL of cDNA was used for qRT-PCR, based on its instructions, on an ABI Prism 7900 Sequence Detection System.

### 4.4. Neutral Lipid Analysis

Intracellular neutral lipid content was determined by BODIPY staining [6]. In this step, 200 μL of cells at approximately 1.5 × 10^6^ cells/mL concentration were washed twice by 0.01 mol/L PBS buffer and incubated with two μL BODIPY 505/515 for 10 min in darkness. After washing with the same volume of 0.01 mol/L PBS, the fluorescence intensity was detected by a microplate reader (Molecular Devices, Sunnydale, CA, USA) with excitation at 480 nm and emission at 510 nm. Meanwhile, the BODIPY stained cells were also observed by a laser scanning confocal microscope (Carl Zeiss, Oberkochen, Germany) with excitation at 488 nm and emission at 550 nm, to visualize the distribution of the intracellular lipid droplets. 

### 4.5. Lipid Extraction and Thin-Layer Chromatography (TLC) 

Total lipids were extracted according to Bligh with a slight modification. Briefly, 10 mg dry cells were incubated with boiling ethanol for 5 min. Cells were disrupted by 0.5 g glass beads (0.5 mm) for 10 min at 4 °C. The cellular lysate was collected. Then, 3 mL of chloroform/methanol (1:2, *v*/*v*) was added with shaking for 1 min, then 1 mL of chloroform and 1.8 mL of ddH_2_O were added with shaking for 20 s. The chloroform layer was transferred to a new tube. Two rounds of addition extraction were performed using 500 μL of chloroform. The chloroform layer was pooled and dried in N_2_, and lipids were dissolved in 100 μL of chloroform. 

Ten μL of lipid samples were spotted onto a TLC plate (silica gel 60, F254) with soybean oil as a control. Chromatography was conducted in hexane/ether/acetic acid (70:30:1, *v*/*v*/*v*) mixture. Lipid bands were visualized by iodine vapor. TAG bands were scraped from the plate and mixed with 1 mL of chloroform/methanol (1:2, *v*/*v*). TAG was then extracted and dried in N_2_. 

### 4.6. Gas Chromatography Mass Spectrometry (GC-MASS) Analysis

A TAG extraction or 5 mg dry cells were used for quantitative analysis. Samples were incubated with 1 mL of 2 mol/L NaOH-CH_3_OH solutions with shaking for one hour. After saponification at 75 °C for 15 min, samples were mixed with 1 mL of 4 mol/L HCl-CH_3_OH and incubated at 75 °C for 30 min. Then, three times, 1 mL of hexane was used for FA methyl ester (FAME) extraction. After filtration, the FAMEs were dried in N_2_ and dissolved in 500 μL of methylene chloride. FAMEs were analysed by GC-MASS according to the previously reported method [6]. The gas chromatography column was VF-23 ms with a size of 30.0 m × 320 μm × 0.25 μm. The Thermo Polaris Q mass spectrometry was equipped with an HP-5MS column, 30 mm × 0.25 mm, film thickness = 0.25 μm. 

### 4.7. Carbohydrate and Protein Analysis

Algae in the stationary phase were harvested and washed with ddH_2_O. Then, cells were weighed after lyophilization. The total carbohydrate was analyzed according to a previous report [37]. Briefly, 0.1 mg dry cells were resuspended in 1 mL ddH_2_O in a tube. Then, 1 mL of freshly prepared 6% phenol and 5 mL of H_2_SO_4_ were added to the tube. The sample was incubated at room temperature for 10 min and 30 °C for 20 min. After proper dilution, absorbance at 485 nm was measured and compared with a standard glucose concentration curve. The protein content was determined using the BCA method. Then, 0.1 mg algal cells were resuspended in 1 mL of 15 mM KH_2_PO_4_ (pH 4.5) and 2 mL of 20% NaOH in a tube. After boiling for 10 min, the tube was centrifuged at 1000× *g* for 10 min. The supernatant was used for protein content determination. 

### 4.8. Transcriptome Sequencing and Data Analysis

Transformant and WT were cultured under the same conditions. The total RNA was isolated by a Takara RNAiso Plus Kit. The mRNA library and paired-end sequencing were performed by Beijing Genomics Institute (Shenzhen, China) using an Illumina HiSeq 4000. The transcriptome was assembled using Trinity and annotated using Trinotate. Differential expression genes were identified using HCE 3.5. They were classified for the categories using the annotation of GO and KEGG pathways with the DAVID. The transcriptomic data have been deposited into CNGB Sequence Archive (CNSA) [38] of China National GeneBank DataBase (CNGBdb) with accession number CNP0003418.

### 4.9. Statistical Analysis

All experiments were conducted in three biological replicates. The data are shown as the mean values. Significance was analyzed by the two-tailed Student’s *t*-test; *, **, and *** represent *p* < 0.05, *p* < 0.01, and *p* < 0.05, respectively. 

## 5. Conclusions

In this study, *crdof* was successfully overexpressed whereas *cracs2* and *cis1* were silenced in *C. reinhardtii*. Total intracellular FAs and TAG elevated remarkably, but starch and protein content decreased, which suggested promoting lipid metabolism and redirecting carbon flux from carbohydrate and protein metabolism pathways. These results were further supported by transcriptome and qRT-PCR analysis of some rate-limiting step genes in lipid, carbohydrate, and protein metabolism. Serving as references, our results provide gene candidates for lipid improvement in other algae species.

## Data Availability

The transcriptomic data generated in this study are available in CNGB Sequence Archive (CNSA) of China National GeneBank DataBase (CNGBdb) with accession number CNP0003418.

## Supplementary Materials

The supporting information can be downloaded at: https://www.mdpi.com/article/10.3390/ijms231710176/s1.
