# Supplementary material for: Increased Lipids in Chlamydomonas reinhardtii by Multiple Regulations of DOF, LACS2, and CIS1
Source: Int J Mol Sci. 2022 Sep 5;23(17):10176. doi: 10.3390/ijms231710176 (PMC9456367; doi:10.3390/ijms231710176)
Supplement: Supplementary file 1 [file ijms-23-10176-s001.zip › ijms-1870605-supplementary.pdf]

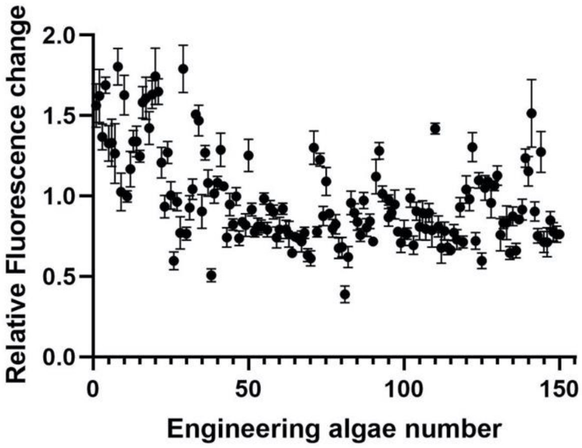

Relative Fluorescence change
2.0
1.5
1.0
0.5
0.0
0
50
100
150
Engineering algae number

A

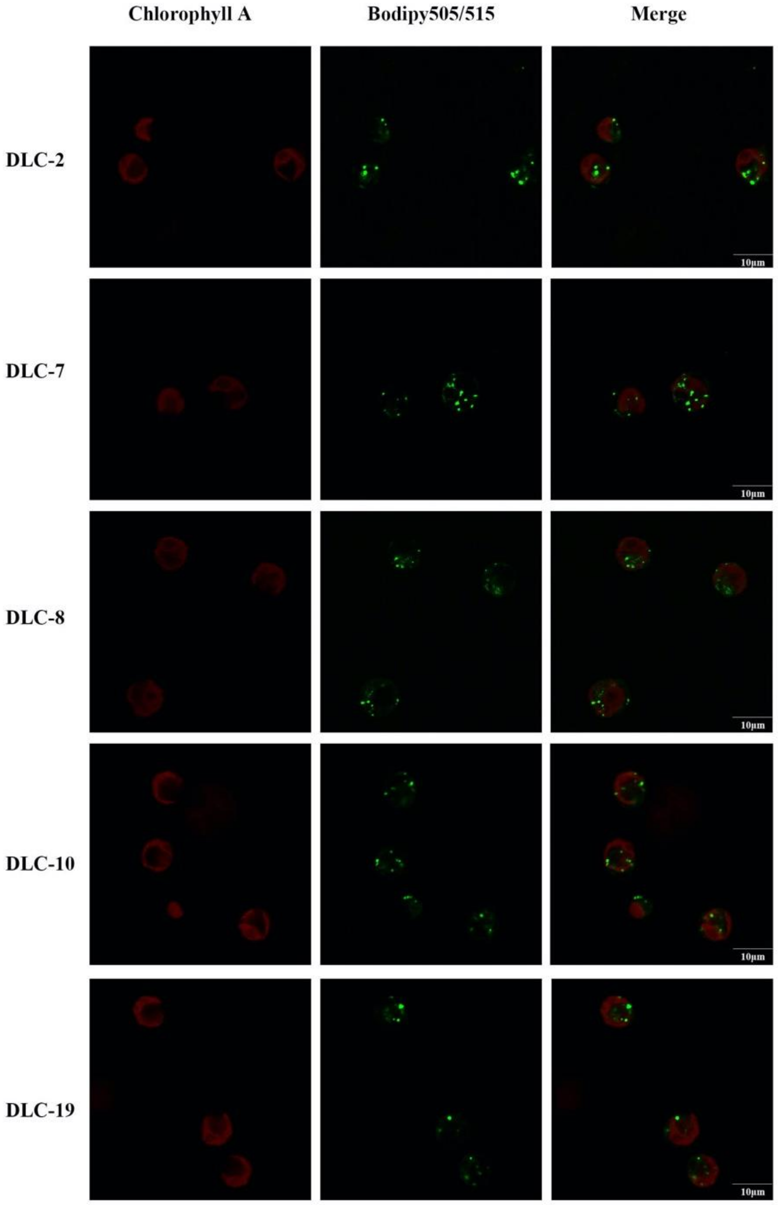

Chlorophyll A
Bodipy505/515
Merge
DLC-2
DLC-7
DLC-8
DLC-10
DLC-19

B

Figure S1. Analysis of intracellular neutral lipids of transgenic strains after staining with BODIPY. A: The results are shown as fold changes in the fluorescence intensities of the transgenic strains compared with that of the wild type strain. Three biological replicates were conducted. B: The microscope images of some strains when they were stained by BODIFY505/515.

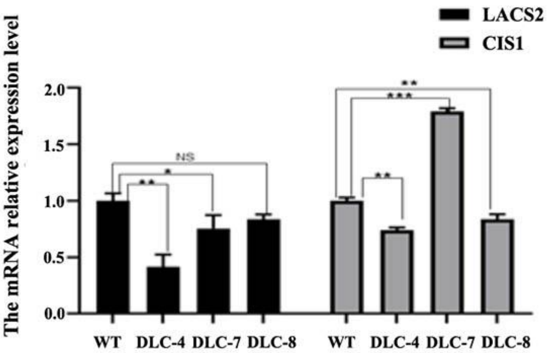

Figure S2. qRT-PCR analysis of the mRNA expression levels of LACS2 and CIS1 in different transformants. Only the mRNA expression levels of CIS and LACS2 in DLC-4 showed decreased patterns.

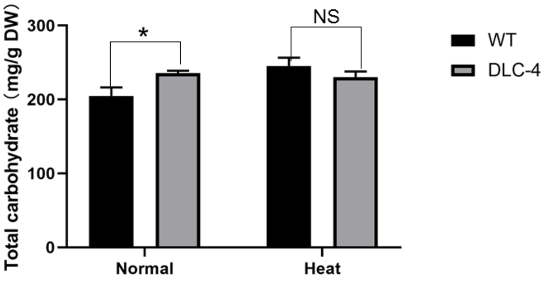

A

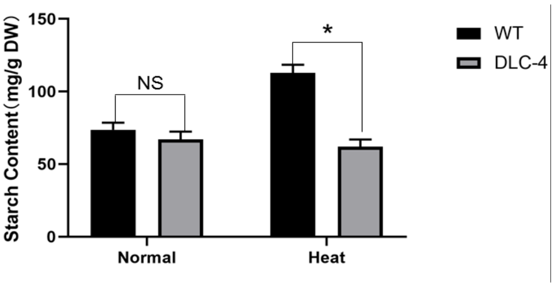

B

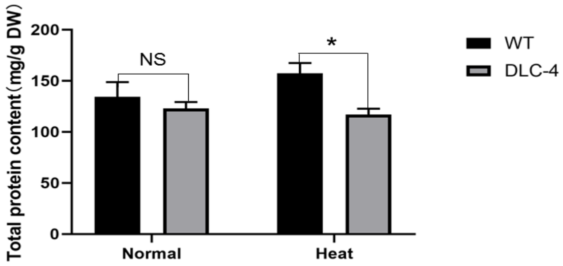

C

Figure S3. Total carbohydrate (a), starch (b) and protein (c) content in DLC-4 and CC849 under normal conditions and after HS. Statistical significance (one-way ANOVA) compared to 0 mM isoprenol is represented by asterisks (* indicates a difference at the $p \leq 0.05$ level).

Table S1. Primers used.

Primers	5'-3'	descriptions
crCIS1F	CAAGTCGCTCACCATGGCGG	Used for crCIS cloning
crCIS1R	CAGTGCAACGTCTGCGAAT	Used for crCIS cloning
LACS-R2	CCGCTCGAGTCAGTGTGTGGCGGGCATTGCGGCGT	Used for LACS cloning
LACS-F2	GGGGTACCATGGCCCCGACAGCGGGG	Used for LACS cloning
RAC-R1	TGGAGTTCATCGCCAACCAC	
RAC-F1	CGTCTGCGACATATCAGTG	
R-ACSF	GCGGCGCAAGCGGGACGACAAGGGGCGGTGGCG	
R-ACSR	ATGGTGAGCGACTTCGATATCCGGGTGGCGGCGGCC	
R-CISF	GGCCGCCGCCACCCGGATATCCAAGTCGCTCACCAT	
R-CISR	GTCCCAAAACAGTGCAACGTCTGCGACATATCAGTG	
A-CF	ACTTGCGACAGAACGCGCCTACAGTGGATGACC	Used for plasmid pMaa7
A-CR	AGCGTAGCGTTAGCGTTAGCGAATTCCCAAAACAGTGCAACGTCTGC	XIR/LACS2-CIS1IR
RA-CF	TTGATCAAGGGCCCGCGCCTACACGTGGATGACC	
RA-CR	TGATAGTGATAGTGATAGTGAGAATTCCCAAAACAGTGCAACGTCTGC	
T1	TGATAGTGATAGTGA	
T2	AGCGTTAGCGTTAGC	
SPR	GTTCTAGTCGCAAGTATTCTAC	Used for transformants screening
SPF	GGGCCCTTGATCCAACCCCTC	Used for transformants screening
ACCDF	AGCATATACGGAGTGCGGTG	
ACCDR	TGCTATACACCTTGCTGCCC	
FAT1F	CATGTATGAGGGCTGCGAGT	
FAT1R	ACCTCACTCATCCCCAGTCT	
DGAT1F	GCATTCGGACGAGGAGCATA	
DGAT1R	CCTGTATCCGTACATGGCCC	Used for qPCR experiments
DGTT4F	AAGCAGGTGGTGAGGGAAAG	
DGTT4R	CACACCACCATTAGGAGGCA	
PAP2F	GCGTGTTTGCCTACTTCCTC	
PAP2R	CACTACTCGCGCCGTACAT	
PDAT1F	GGGTATAGGGGGAAGGGGTT	
PDAT1R	CCTGCAATCAATCTCGCTGC	
